# Designing nanoparticles for delivery of neurotrophic proteins

**DOI:** 10.1186/1750-1326-8-S1-O16

**Published:** 2013-09-13

**Authors:** Angelina Angelova, Borislav Angelov, Sylviane Lesieur

**Affiliations:** 1 CNRS UMR8612 Institut Galien Paris-Sud, Univ Paris Sud 11, Chatenay-Malabry, France; 2 Institute of Macromolecular Chemistry, Academy of Sciences of the Czech Republic, Prague, Czech Republic

## 

Nanoparticulate systems for neurotrophic factor delivery are currently studied in an attempt to solve some of the challenges in neurodegenerative disease treatment. Nanomedicine for brain disorders has faced difficulties in cerebral administraton of fragile neurotrophic proteins and high costs. According to recent studies, the signaling protein brain-derived neurotrophic factor (BDNF), being a main mammalian neurotrophin, is regarded as a therapeutic target for a number of neurodegenerative and psychiatric diseases (Huntington’s disease, Alzheimer’s disease, Parkinson’s disease, amyotrophic lateral sclerosis, depression, schizophrenia, *etc*)*.* However, a beneficial BDNF treatment is still not clinically available for patients. Thanks to their safety and feasibility for large-scale production, lipid nanoparticles (NPs) and nanostructured vehicles are considered as key candidates for delivery of BDNF-based therapeutics. Nanoparticles may serve as reservoirs for controlled drug release and may influence the biodistribution and bioavailability of the administered protein. Nanoencapsulation in lipid particles provides protein stabilization against degradation and a possibility for targeted delivery. Lipid nanostructure types and liquid-crystalline phase transformations are suggested to govern the cell uptake mechanisms. Fusogenic lipids, like the cubic-phase forming monoolein, characterized by pore-inducing propensity and significant structural influence on biomembranes, have drawn the attention in designing BDNF delivery vehicles. Nanoparticles were prepared from self-assembly mixtures of lipids and BDNF and functionalized by stabilizing amphiphilic polymer derivatives. The resulting nanoscale organizations were revealed by cryogenic transmission electron microscopy (cryo-TEM) and X-ray structural analysis (SAXS) in order to evaluate the ability of the investigated particles for protein upload. The interaction of differentiated human neuroblastoma SH-SY5Y cells (a cellular model of neurodegeneration) with lipid nanocarriers of BDNF was studied by means of confocal fluorescence microscopy imaging. The obtained nanoparticles, encapsulating neurotrophic protein, may be anticipated to show therapeutic potential in repairing damaged neurons by regulation of the neuronal survival and plasticity.
